# A simulated “Night-onCall” to assess and address the readiness-for-internship of transitioning medical students

**DOI:** 10.1186/s41077-017-0046-1

**Published:** 2017-08-14

**Authors:** Adina Kalet, Sondra Zabar, Demian Szyld, Steven D Yavner, Hyuksoon Song, Michael W Nick, Grace Ng, Martin V Pusic, Christine Denicola, Cary Blum, Kinga L Eliasz, Joey Nicholson, Thomas S Riles

**Affiliations:** 1 Department of Emergency Medicine, NYU School of Medicine, New York, New York USA; 2 New York Simulation Center for the Health Sciences, New York, New York USA; 3 Institute for Innovations in Medical Education, NYU School of Medicine, New York, USA; 4 Department of Emergency Medicine, Center for Medical Simulation, Institute for Medical Simulation, Harvard Medical School, Boston, MA USA; 5 Health Science Library, NYU School of Medicine, New York, New York USA; 6 Program for Medical Education and Technology (PMET), NYU School of Medicine, New York, USA; 7 Department of Journalism, Central Connecticut State University, New Britain, CT USA; 8 Department of Education, Georgian Court University, Lakewood, NJ USA; 9 Department of Surgery, NYU School of Medicine, New York, USA; 10 Department of Medicine, Division of General Internal Medicine and Clinical Innovation, New York, New York USA; 11 Research on Medical Education Outcomes (ROMEO) Unit, Program for Medical Education Innovation and Research (PrMEIR), NYU School of Medicine, OBV CD-401, 462 1st Avenue, New York, New York 10016 USA

**Keywords:** Transitions to residency, Immersive simulation, Mixed modality experiences, Educational experience, Team work, Basic clinical skills, Communication between team members, Handoffs, Oral presentations, Readiness-for-internship assessments, Competency-based medical education, Entrustable Professional Activities

## Abstract

Transitioning medical students are anxious about their readiness-for-internship, as are their residency program directors and teaching hospital leadership responsible for care quality and patient safety. A readiness-for-internship assessment program could contribute to ensuring optimal quality and safety and be a key element in implementing competency-based, time-variable medical education. In this paper, we describe the development of the Night-onCall program (NOC), a 4-h readiness-for-internship multi-instructional method simulation event. NOC was designed and implemented over the course of 3 years to provide an authentic “night on call” experience for near graduating students and build measurements of students’ readiness for this transition framed by the Association of American Medical College’s Core Entrustable Professional Activities for Entering Residency. The NOC is a product of a program of research focused on questions related to enabling individualized pathways through medical training. The lessons learned and modifications made to create a feasible, acceptable, flexible, and educationally rich NOC are shared to inform the discussion about transition to residency curriculum and best practices regarding educational handoffs from undergraduate to graduate education.

## Introduction

*“It still doesn’t quite feel like I am able to jump in and start on July 1…the nurses expect you to be the doctor, the patients expect you to be the doctor, your colleagues expect you to be the doctor”.*

*~4th year medical student 2 weeks before graduation expressing anxiety about transitioning to residency.*

*“We get to see July 1st as medical students and get to see how a lot of Interns really struggle with some basic skills”.*

*~3rd year medical student a year before graduation voicing concern about transitioning to residency.*

Medical students transitioning from undergraduate medical education (UME) to graduate medical education (GME, also referred to as “residency” or “internship”) experience uncertainty and distress about their readiness-for-internship [1–3]. This lack of readiness may be partially responsible for the “July effect”—a reported increase of 10% in fatal medical errors in teaching hospitals in North America when these new graduates enter the workforce each July [4]. Residency program directors are just as anxious about integrating the incoming medical students into a fast-paced and complex health care system because they are aware that clinical experience and competence during the senior year of medical school is variable, both within a single school and across institutions [5–7], and a new resident class is typically made up of graduates of many medical schools. This heterogeneity in readiness has led residency programs and hospital leadership to implement orientation programs and increase supervision to ensure patient care quality and safety as new trainees learn to function effectively in their latest roles [8, 9]. Some medical schools have also implemented transition courses; however, these are generally focused by clinical discipline [10]. A clinical discipline-agnostic readiness-for-internship program, administered just prior to medical school graduation, would serve many important purposes including (1) preparing near-graduate medical students for a smooth and safe transition to residency, (2) building an assessment program with the intention of ultimately benchmarking and reporting readiness-for-internship metrics, regardless of clinical discipline, and (3) providing a meaningful educational handoff between UME and GME in the USA and beyond.

**Fig. 1:**
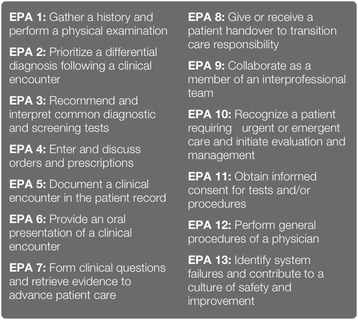
The 2016 NOC activities were tailored to capture and assess the 13 core EPAs for medical students transitioning to residency. For a complete version of the Core Entrustable Professional Activities for Entering Residency please go to: www.mededportal.org/icollaborative/resource/887

A competency-based readiness-for-internship assessment program is both timely and critical to the UME-GME continuum [10].In recent years, patient safety and quality assurance committees of hospitals and residency program directors have been called upon by accrediting agencies, malpractice insurance companies, and the general public to demonstrate that trained residents are capable of providing the level of care for which they have been assigned. Residency Review Committees, the clinical discipline specific accreditation bodies of the US Accreditation Council for Graduate Medical Education (ACGME), have provided guidelines outlining what a first-year resident can and cannot do without direct supervision until competency has been documented [11]. In 2014, the Association of American Medical Colleges (AAMC), responsible for accrediting medical schools in the USA, released a set of 13 core Entrustable Professional Activities (EPAs) for entering residency (Core EPAs) (see Fig. 1). EPAs are units of professional practice a trainee can be trusted to accomplish unsupervised once he or she has demonstrated sufficient and specific competence. Authors of the core EPAs provided detailed guidance meant to drive the community toward refining, measuring, and benchmarking the *minimal level of competence* expected of a medical school graduate [12]. As of yet, there is little consensus on *how to assess* the Core EPAs of new residents or *what type* of transition documentation (or “handoff”) to residency programs would be meaningful [13, 14].

Although ensuring readiness-for-internship is challenging, there are unacceptable negative consequences for patients, institutions, programs, and for the individual professional if “onboarding” is not done effectively. Simulation has a critical role to play in both reducing the risk of iatrogenic harm to patients [15, 16] and assessing fundamental clinical competence critical to creating an institutional culture of safety [17–20]. Ideally, with the implementation of a meaningful simulation-based assessment program just prior to medical school graduation, actionable formative feedback can be provided to both the learner and GME Program Directors to achieve these goals.

In this paper, we describe in detail the development of a complex, immersive simulated, Night-onCall (NOC). We believe that NOC is an innovative program for a number of reasons including the fact that it (1) was designed iteratively and in response to specific local needs and evolving research questions, (2) it can be reproduced at most medical schools without need of sophisticated simulation facilities, and (3) it provides *both* an authentic educational experience for and is likely to enable high value core EPAs assessment of transitioning medical students.

## Developing *NOC*

### Conceptual framework underlying NOC

NOC is a multi-station experience in an Objective Structured Clinical Exams (OSCEs) format [21–23]. Since the 1960’s OSCEs utilizing standardized (a.k.a. “programed,” “simulated”) patients to assess core clinical skills have become a ubiquitous part of medical education assessment programs—used worldwide in a vast array of formats and for a variety of purposes including physicians’ licensing examinations. NOC aligns with literature that supports the utility of a well-designed OSCE as an assessment of clinical competence, assuming careful attention is paid to “contextual fidelity,” which includes the interprofessional nature of most medical work and accurate “professional role reproduction” [24]. NOC is the current focus of a research program in which we explore the measurement of clinical competence for the purpose of supporting increasingly individualized pathways through medical training [25].

### The team

NOC was developed by a multidisciplinary and inter-professional team consisting of physician, nurse, medical librarian, and PhD-prepared educators from Emergency Medicine, Internal Medicine and Surgery and Obstetrics and Gynecology. Our team, as whole, has extensive expertise in using simulation in undergraduate and graduate medical and nursing education.

**Fig. 2:**
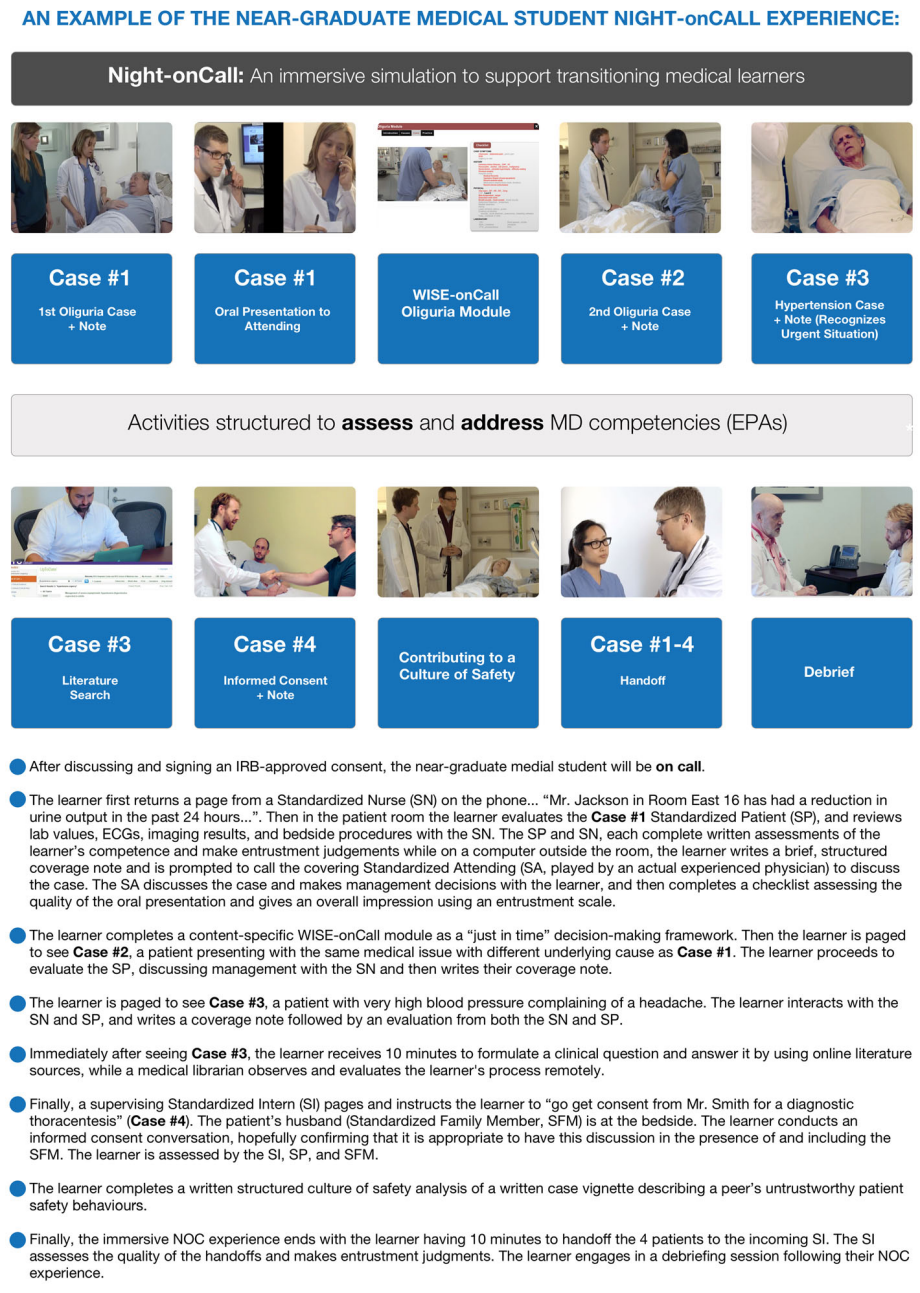
The Night-onCall experience from the student’s perspective

Table 1 details how we incrementally developed NOC over a 3-year period into a complex multi-modal, immersive simulation and a summary of our experience. The individual components of the 2016 NOC experience were refined and designed to address and assess each of the 13 Core EPAs. Fig. 2 illustrates what a medical student would experience in the 2016 iteration of NOC.

**Table 1:** Development of the Night-onCall (NOC) event

Development year over year	2014	2015	2016 (Night-onCall)
Clinical cases/mixed modality	*Case 1: Oliguria* “I am calling about Mr. Jackson, 64-year-old man S/P Elective endovascular repair of AAA, post-operative day 3. His urine output has dropped and he has mild abdominal pain” (Has urinary retention, BPH). *WISE-onCall module with 3 practice cases.* *Case 2: Oliguria* “I am calling about Mr. Taylor. 57-year-old man here for observation to rule out acute cardiac ischemia and pulmonary embolism. His urine output has dropped and he remains without chest pain” (received contrast for a cat scan).	*Case 1: Oliguria (same).* *WISE-onCall module with three practice cases.* *Case 2: Oliguria (same).* *Case 3: headache* “I’m calling about Mr. Johnson, 64-year-old man S/P a AAA repair day 3, and is complaining of a severe headache” (has a blood pressure of 195/99 and a history of HTN). *Case 4: headache:* “Hi. Are you covering for Mr. Kolinsky, 62-year-old man S/P internal fixation of an ankle fracture…I wanted to let you know that he is having a severe headache” (history of migraines on propranolol for prevention). Form *clinical question and retrieve evidence* to advance clinical care. *Culture of safety analysis of a paper case*: vignette describing pre-entrustable peer on internal medicine clerkship-structured response identifying evidence of behaviors and assessment of entrustment. *Handoff all two cases* to fellow intern (standardized): prioritize based on urgency. Assessment of entrustment.	*Case 1: Oliguria (same).* Plus: oral presentation to attending. *WISE-onCall module with three practice cases.* *Case 2: Oliguria (same)* *Case 3r: headache (revised)* “Hi. Are you covering for Mr. Brooks, 60-year-old man being treated for Diverticulosis, …I wanted to let you know his blood pressure is really high” (195/99 currently, non-focal neuro-exam, history of migraine headaches on propranolol for prevention)? Form *clinical question and retrieve evidence* to advance clinical care (same). *Case 4r: Go “get” consent (revised)*: “Hi. This is Randy, your second year resident. You are covering Mr. Smith a 40 y/o with a cough, fever and pleural effusion. You need to go consent him for a thoracentesis. I will meet you at the bedside in 1 h.” (The resident will explain the procedure if asked, patient’s husband is in the room). *Culture of safety analysis of a paper case: same.* *Handoff all four cases* to fellow intern (standardized): prioritize based on urgency. Assessment of entrustment.
Number and types of participants	52 4th-year graduating medical students.	66 4th-year graduating students. 42 3rd-year students (rising seniors).	89 students. 35 4th-year, 12 3rd-year accelerated, 36 3rd-year, 65-year pathway.
Event length	3 h/student, Over 3 full days in simulation center.	3 h/student, over 9 full days in simulation center.	4 h/student, over 16 half days in simulation center.
Incentive	$100/Student, IRB-approved protocol.	$100/Student, IRB-approved protocol.	$100/student, IRB-approved protocol.
EPA’s addressed and assessed	1–5, 9,12	Piloted oral presentation, handoff, evidence-based medicine, culture of safety. 1–10, 12–13	1–13
Study questions	In what ways are our near graduates ready for internship? Does WISE-OnCall “just in time” improve core clinical skills required for common clinical coverage issues? Do different forms of feedback (short-form checklist vs. whole-form checklist) provided during the practice cases have an impact on learning outcomes?	Does simulated clinical exposure before WISE-onCall enhance learning from it? Does WISE-onCall improve clinical performance in content discordant cases? Exploratory: 3rd-year vs. 4th-year students? Which core EPAs for entering residency can we reliably assess in an integrated authentic simulated experience?	Is it feasible to assess all core EPAs for entering residency in an integrated authentic simulated experience? What are the differences in readiness for residency among clinically experienced students in different curricular pathways?
Measurements (assessor: assessed domains**)**	SP: Communication skills (data gathering, rapport building, patient education and counseling), history gathered, physical exam, professionalism, recommendations (entrustment equivalent). SN: collaboration, inter-professional communication, rapport building, professionalism (entrustment). Patient note: reporter, interpreter, manager, clinical reasoning. Faculty: clinical reasoning, entrustment. Structured domain specific medical knowledge (clinical schema).	*Plus* Paper case: culture of safety: entrustment of peers. Medical librarian: ability to formulate answerable clinical questions and identify a literature based answer. Peer: handoff quality and/entrustment.	*Plus* Faculty: oral presentation skills and entrustment. Case no. 3: SP/SN: recognize a patient requiring urgent or emergent care and initiate evaluation and management. Case no. 4: SP/standardized resident/spouse: ability to perform an ethical and legal informed consent discussion and effectively include family members.
Feedback, findings and remaining questions	• Students appreciate the opportunity to practice and learn before July 1. • WISE-onCall module is useful “just in time”. • Students question authenticity of working with nurse in patient’s room. • Extreme variability in measured “readiness” and sophistication in clinical schema. • Majority of students improved significantly after WISE-onCall (some did not). Does this reflect readiness for learning from clinical cases? • Simpler forms of feedback with in Wise onCall are as effective as more complex ones (RCT). • Although they need to be refined, our assessments were reasonably reliable, authentic and synthetic. • Many of the common topics required for transition from UME to GME can be assessed and addressed using this style of blended assessment/learning experiences.	• All clinical students (3rd and 4th) appreciate the practice and authenticity. • Educational utility is high. • Students demonstrate the best clinical skills and clinical reasoning after they complete an SP/SN case on the same topic before a Wise onCall module (neither alone is enough). • MS 3s have *more comprehensive* basic clinical skills than MS 4s. • Both MS 3s and 4s get a significant *boost* in content specific structured knowledge from blended WISE-OnCall and simulation experience. • 4th-year students gained more in the domains of clinical management and overall clinical reasoning than the 3rd-year students. • This may be secondary to boosting effect of the experience on knowledge and skills they had obtained but forgot. • Almost all students recognize pre-entrustable “culture of safety” behaviors in a peer and can recommend strategies to address these. • The quality of ability to formulate answerable clinical questions and identify a literature-based answer is highly variable.	• Continued enthusiasm for high educational yield of the event. • Feasible to assess all 13 core EPAERs confirmed. • No significant differences among students in accelerated MD program and traditional 4th-year program (small sample). • Attendings impressed with variability in intern readiness based on oral presentation. • Both competency measures and entrustment measures can be made. • What should we do with students who perform poorly on NOC? • What would be the more useful design for educational handoffs from UME to GME? • Can we establish predictive models and cut offs for the data produced in NOC?

### *Types of* NOC *assessments*

#### Web-based multimedia module

In response to the increasing focus on medical students’ readiness for residency, and based on 10 years of experience building and studying WISE-MD—a web-based core surgery clerkship curriculum [26], our team created WISE-onCall—a set of web-based, multimedia modules targeted at enhancing the ability of novices to address common clinical coverage issues. The modules are designed as a cognitive apprenticeship framework [27], starting with two “partially worked” case examples including video demonstration of inter-professional interaction, utilizing the instructional strategies of modeling, coaching, scaffolding, and fading of instructional guidance and then three text-based practice cases where the learner applies diagnostic skills and obtains feedback. To date, eight WISE-onCall modules have been completed with plans to build at least five more in the next 2 years [28]. For NOC, we selected the Oliguria (low urine output) WISE-onCall module because it is a topic that all students are likely to have basic familiarity with by the end of medical school and it is a condition interns in all clinical disciplines can expect to encounter during a typical night on inpatient call. (Addresses EPAs #1,2,3,4,9,10,12).

#### Performance-based assessment (PBA)

Initially, in 2014, we designed two standardized patient (SP) and standardized nurse (SN) cases of relatively equal difficulty (case no. 1, case no. 2) for pre and post of the WISE-onCall module. In 2015, we developed two additional SP/SN cases, (case no. 3, case no. 4) in order to explore how clinical case content concordance and sequencing of PBA and WISE-onCall, impacted performance across the simulation activities [31].In 2016, we revised case numbers 3r and 4r to enable us to address, assess, and align PBAs to the core EPAs (see Table 1 for details).

Learners’ clinical skills including inter-professional teamwork were assessed using SP/SN completed checklists developed based on extensive/prior research [22]. Clinical reasoning was assessed based on student-completed patient coverage notes and scored by a clinician based on a rubric [29, 30]. Rigorous methods were employed to develop SP/SN roles and checklists, as well as recruit, train, and calibrate actors for both case portrayal (3 h) and rater reliability (3 h) [23] (Addresses EPAs 1,2,3,4,5,9,10,12).

#### Oral presentation

Experienced physicians from the study team played the role of a standardized attending (SA) for case number 1. The SA received a phone call from the study participants following the case number 1 clinical encounter. A detailed guide to the case and the task were provided. Specifically, the guide included the clinical details of the case and a set of standardized prompts to be used to encourage the learner to share their clinical reasoning and establish a management plan. The SA was also responsible to assess the quality of the oral presentation using a checklist designed based on the detailed description of core EPA no. 6 [31] and make an entrustment judgment.

#### Evidence-based medicine activity

Following case no. 3 learners, seated in front of a computer with an Internet access, were given 10 min to define a clinical question based on this case and instructed to use Web-based resources to find the best answer to a clinical questions provided to them (e.g., “What is the best initial management for urgent hypertension?”). A computer program was used to allow a medical librarian to remotely observe the learner’s progression through the activity both in real time and based on a recording. Using this approach, the medical librarian was also able to assess the learner’s ability to formulate a clinical question and use digital resources to identify high quality evidence to guide the patient’s care as described by EPA no. 7.

#### Patient handoff

We recruited senior medical students to play the role of the standardized intern (SI) taking over the clinical service. Each SI was trained to use a structured evaluation instrument, modified from a published instrument, to assess the quality of the handoff [32] as well as provide an entrustment judgement (EPA no. 8).

#### Culture of safety exercise

Participants were first given time to read a detailed vignette describing a pre-entrustable intern’s approach to a series of common quality and safety challenges on an inpatient ward [31]. Then, in written responses to open-ended prompts, the participants listed both the interns’ behaviors and attitudes that interfered with a culture of safety and suggested actions needed for systems improvement. A faculty member (GN) assessed students’ written responses based on a rubric designed based on the description of the AAMC’s EPA no. 13 [33].

### Recruitment of students

For all phases of NOC, we recruited near-graduate medical students by email. If the student agreed to participate, he or she could sign up for a scheduled slot in the simulation center by clicking on a Universal Resource Locator (URL) embedded in the recruitment email. Study staff then confirmed the date with the participant and provided him/her background information regarding the study via email. Participation was entirely voluntary, written informed consent was obtained and a financial incentive was provided.

## Resources needed to implement NOC

The NOC experience was hosted by the New York Simulation Center [34]. We estimate our cost-per-student for NOC to be around $500 (US). This includes SP/SN salaries and staff time for planning and running the event, including SP training, student recruitment, and scheduling. This estimated cost does not include a facility fee, the study incentive, case development time, patient note scoring, data entry and management, and physician preceptor time.

### Lessons learned

In building this experience, we have learned many lessons that may be of interest to other’s seeking to build similar assessment events. While currently we do not share any assessment data with students, ultimately, we seek to use the competency assessments and entrustment judgments for feedback to students on their readiness and as a handoff to residency training program directors. The following is what we learned so far.

#### NOC is feasible

As we have demonstrated, it is feasible to host a NOC for a large number of students. However, this can only be done with championship from leadership, adequate funding and committed professional and administrative personnel. The team met weekly for the 3 years; it took to develop materials, pilot, and refine the program. The staging of the full NOC event required several months of planning which included scheduling space, recruiting and training actors, faculty and students playing roles, and recruiting and scheduling participants. Data entry, cleaning, analysis, and interpretation also required adequate resources. Advanced simulation facilities or equipment were not required to host the NOC.

#### NOC is acceptable

NOC is an immersive, complex, mixed-modality simulation experience, aimed at creating an authentic opportunity to rehearse being an Intern “on call.” Although it will require more work to establish the program as an effective means of measuring students’ readiness-for-internship for high-stakes purposes, the participants routinely expressed in debriefing portion of the NOC experience, that it helped them better understand their readiness and identify knowledge/skill gaps prior to their transition.

#### NOC is a flexible structure

Depending on local needs and resources, there are ways to modify the program to reduce cost and shorten the time needed, while at the same time, still achieving the same objectives. Based on our experience, we believe the EPA framework allows for a great deal of creativity and innovation. For instance, we choose to assess EPA no. 13 using a written assessment of a paper case rather than a complex simulation others have used [35]. Other schools prepare students for a night on call by integrating assessments into their required advanced clerkships [36]. In the future, we plan to conduct head-to-head comparisons of various strategies to better understand relative educational and assessment value and costs.

#### NOC *will likely produce valuable information*

One goal of the analysis of our experience and data is to understand the educational value of the components of NOC. From the point of view of the students who have volunteered to participate, this low-stakes experience was almost uniformly seen as time well spent, educational, and anxiety-reducing. This may change as we refine the competency measures and entrustment judgments and start providing detailed feedback. At our school, 12 years ago, we established a Comprehensive Clinical Skills Exam (CCSE), an 8-station Objective Structured Clinical Exam, to serve as a final performance exam for the core clinical clerkships. Similarly, we developed the CCSE as an assessment *for* learning or formative experience where it was very popular with and very much appreciated by students. Once we transitioned the CCSE to an assessment *of* learning, (as defined by van der Vleuten et al. [37]) or summative, high-stakes experience where students were required to pass the exam, its popularity and the enthusiasm among students decreased. We suspect this may be an inevitable trade-off for some students, but we do hope to engage students in embracing the value of the data produced by NOC.

#### Next steps for NOC

We are currently experimenting to find effective ways to visualize the NOC data and report it to students for the purposes of guiding them in preparation for internship. Despite desiring educational handoff information on their incoming interns, residency program directors are suspicious of assessments done in the undergraduate setting and do not yet “trust” evidence of readiness [1]. With this in mind, we are exploring how, if at all, residency program directors would find this type of performance data useful to plan supervision during the transition months, given that in the USA, they are contractually committed to training incoming residents at the time of medical school graduation.

We are also exploring both how best to understand the entrustment judgments generated in NOC [38, 39] and adding self-assessment measures (e.g., context-specific self-efficacy, affect, and cognitive load relevant measures) to examine the value of experiences like NOC on understanding a student’s metacognitive capabilities [40]—thought to be crucial to the lifelong learning required by a career in medicine in the twenty-first century.

#### Is NOC a valid approach to enhance readiness for internship?

We embraced the complexity and context-based nature of competence in building NOC. As a consequence, it will require a great deal of work to establish validity and set standards with the NOC outcome data for the purpose of high-stakes promotion decisions. Our team is currently working toward this goal. NOC’s design is grounded in both a conceptual (situated mixed modality clinical experiences in an immersive simulation) and content framework (core EPAs), created through national consensus and endorsed by the AAMC. When available, we based our assessment instruments on tools with previously reported internal validity data and we are working to ensure there is acceptable reliability to all our assessments. NOC balances the difficulty of having highly reliable measures with the fact that we are generating a large number of assessments on each student from a variety of perspectives (patient, nurse, expert, peer—a simulated 360° workplace assessment. We plan to follow some of our subjects forward into the first year of residency and beyond to see if strengths and weaknesses identified during the NOC experience are associated with adjustment to internship and demonstrated skills, and, in the longer run, to study if NOC predicts success in residency training and beyond.

The NOC program has already resulted in curriculum changes. For example, our clerkships and sub-internships have incorporated WISE onCall modules and related exercises that address clinical reasoning and provide examples of professional behavior, teamwork, and communication.

## Conclusion

If the AAMC EPAs are to become the standard by which we assess transitioning students’ preparedness for residency, we will need to assure all of our students reach those standards and continue to be able to perform at that level at the time they are transitioning to graduate level medical education. Building programs, like NOC, will also enable medical schools to move toward a competency-based, time-variable curriculum that many now believe is the best way forward [41–43]. We have described a program for achieving these goals that is feasible, acceptable, flexible, and likely to produce valuable information for learners, educational leaders and policy makers.
